# Giant leiomyoma management during elective cesarean section: a case report

**DOI:** 10.11604/pamj.2023.45.73.39357

**Published:** 2023-06-02

**Authors:** Ottavio Cassardo, Giuseppe Perugino, Francesco Nesa, Manuela Wally Ossola, Enrico Ferrazzi, Francesco D´Ambrosi

**Affiliations:** 1 Unit of Obstetrics, Department of Woman, Child and Neonate, Mangiagalli Center, Fondazione IRCCS Ca' Granda Ospedale Maggiore Policlinico, Milan, Italy,; 2 Division of Pathology, Fondazione IRCCS Ca' Granda Ospedale Maggiore Policlinico, Milan, Italy

**Keywords:** Cesarean myomectomy, leiomyoma, uterus exteriorization, case report

## Abstract

We present the unique case of a 44-year-old gravida 3 para 2 woman with complaints of monolateral perception of fetal movements who underwent elective cesarean section and hysterectomy for the presence of an exceptionally voluminous infralegamentary leiomyoma. Cesarean section required in-depth preoperative planning and was possible only after gravid uterus exteriorization. Myomectomy and hysterectomy were then necessary to reestablish the physio-anatomical pelvic environment. The patient was discharged after regular and uncomplicated postoperative time. In recent years, the paradigma of avoiding cesarean myomectomy due to fear of hemorrhage has been questioned by many authors and in certain cases cesarean myomectomy may even be undeferrable. We describe an innovative surgical technique which could be useful to obstetricians approaching similar uterine masses during cesarean sections.

## Introduction

**Figure 1:**
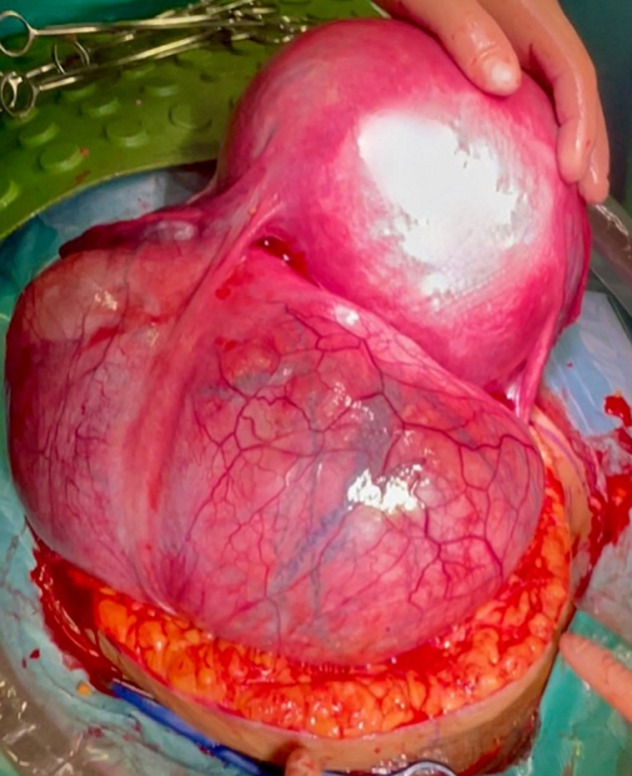
exteriorization of the leiomyoma (right) and the gravid uterus (left)

Leiomyomas are common benign tumors of the reproductive tract in women of childbearing age. Their exact incidence in pregnancy should take into account maternal age at conceivement, obesity and race among other factors. Reported prevalence ranges between 2% to 4%, but recent evidence is showing higher figures [1]. Myomectomy during cesarean section is traditionally avoided due to the increased vascularization of the pregnant uterus, leading to possible massive hemorrhage and consequent, avoidable, intra-cesarean hysterectomies [2]. However, in modern obstetrics, with advancements in anesthesia, adequate availability of blood products, selective devascularization techniques and a multidisciplinary approach, myomectomy has returned to be a possible option during cesarean sections, to save the patient from future morbidity due to multiple surgeries, anesthetic complications and costs [3]. Moreover, in certain cases, leiomyomas might reach exceptional volumes because of hormonal stimulation that occurs in pregnancy; in these instances, cesarean-myomectomy may even be undeferrable. Here, we report the case of a successful intra-cesarean myomectomy of a “giant” leiomyoma and following necessary hysterectomy without any complications.

## Patient and observation

**Patient information**: a forty-four-year-old gravida 3 para 2 North African woman at 34 weeks of gestation presented at our Emergency Unit complaining of heavy abdominal pain and a protruding right abdominal mass, reporting perception of fetal movement limited to the contralateral, left, abdominal side.

**Clinical findings**: the patient had already been admitted at our Maternity Hospital at 32 weeks of gestation with painful uterine contractions; ultrasound (US) evaluation revealed the presence of a single, right-round ligament leiomyoma of 25 x 20 centimeters with a left-side uterine and cervical dislocation. She was under labetalol for chronic hypertension and low dose ASA for high risk of preeclampsia at the first trimester screening.

**Diagnostic assessment**: she underwent tocolysis and fetal lung maturation and was dismissed. Elective cesarean section was planned at 36 weeks of gestation, given the position of the leiomyoma. At second admission the leiomyoma was much larger than at 32 weeks. Fetal growth and movements were normal at US. Amniotic fluid index was 7 cm. There was no associated vaginal bleeding. She tested positive for SARS-CoV-2 nasopharyngeal swab and tested negative after seven days. She was therefore hospitalized for caution. On first day of hospitalization, a second level ultrasound examination confirmed the presence of a 30 x 20 x 18 centimeters right-round ligament leiomyoma, with complete dislocation of the uterus from the pelvis towards the left upper abdominal quadrant, just underneath the left hemithorax.

**Diagnosis**: her case was discussed on a multidisciplinary level, with obstetric surgeons, expert ultrasonographists and anesthesiologists, and the decision of intra-cesarean myomectomy was taken; cesarean hysterectomy appeared to be likely either for massive hemorrhage or for uterine anatomical distortion. Informed consent was obtained for these procedures by the patient, who expressed the will for tubal ligation.

**Figure 2:**
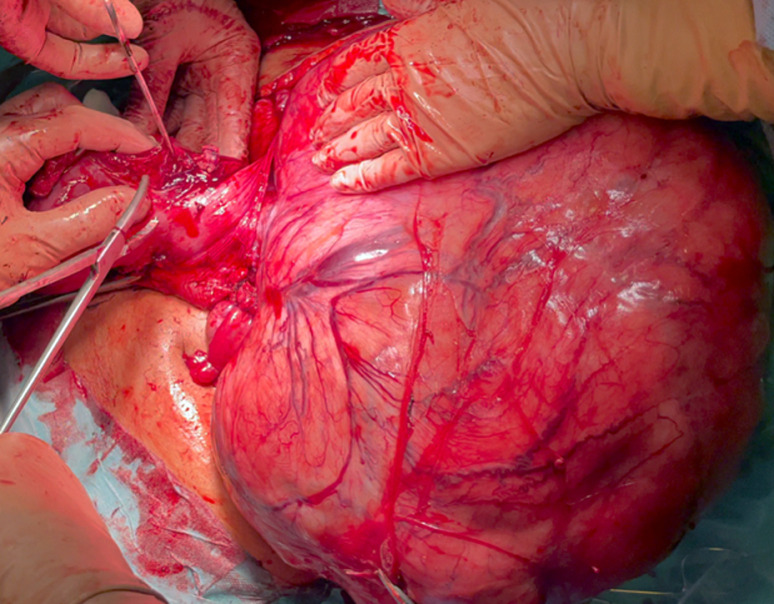
myomectomy: preparation of the leiomyoma vascular pedicle

**Therapeutic interventions**: on the due day, at 36 weeks of gestation, her heart rate was 94 beats per minute, blood pressure was 135/85 mmHg. No uterine contractions were registered at the preoperative cardiotocography nor in the preceding days. Vaginal examination depicted a non-dilated nor effaced cervix; evaluation of the presenting part was not possible due to the obstructing leiomyoma. Her preoperative hemoglobin was 11,8 gr/dL, hematocrit was 38.2%, PCR 0.77 mg/dL and she had regular coagulation tests; her blood group was 0 positive. Adequate blood products were arranged. General balanced anesthesia was induced, and the patient was intubated. Abdominal access was obtained with an infra-umbilical bisiliac incision of 20 cm. Fascial planes were developed up to the umbilicus on both sides. On peritoneal opening, the abdomen was entirely occupied by the fibroid mass whereas the uterus was left- and upward-rotated to the splenic lodge and therefore not accessible for safe hysterotomy. The decision of exteriorization of the gravid uterus was taken. The uterus was initially mobilized even more toward the left-thorax, in order to liberate and exteriorize the uterine mass. Exteriorization of the gravid uterus was then possible with care taken on left-round ligament tension (Figure 1). These maneuvers were performed with continuous feedback on hemodynamic changes by anesthesia. A vertical, corporal, uterine incision was performed, and a 3.1 kg male baby was delivered. 1-minute-delayed umbilical clamping was performed. APGAR score at 1 and 5 minutes was 9 and 10. After assisted placental expulsion, the uterine wound was closed with a monofilament suture. As the mass was bulging into the incision line and heavily distorting the anatomy of the uterus, planned myomectomy was confirmed. We started by dissecting the right round ligament and opening of the anterior leaflet of the broad ligament to liberate the mass (Figure 2). Careful fibroid enucleation was performed with bipolar forceps after identification of the right ureter. Myomectomy was completed after preparation and dissection of the vascular pedicle of the mass, on the right side of the uterine isthmus. A complete hemostasis was achieved. On surgical gross examination, the uterine anatomy could not be reckoned, and heavy intramural right hemorrhage was evident. Total hysterectomy was then performed along with bilateral salpingectomy as for the will of the patient. The total duration of surgery was 113 minutes, the amount of blood lost was around 700 mL. Broad spectrum antibiotics were administered in the postoperative days.

**Figure 3:**
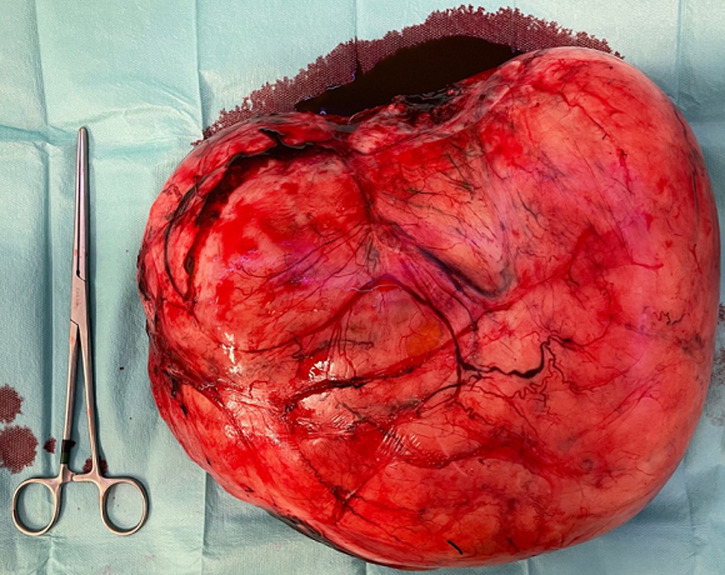
gross specimen: 6.7 kg and 28 x 11 x 13 centimeters

**Figure 4:**
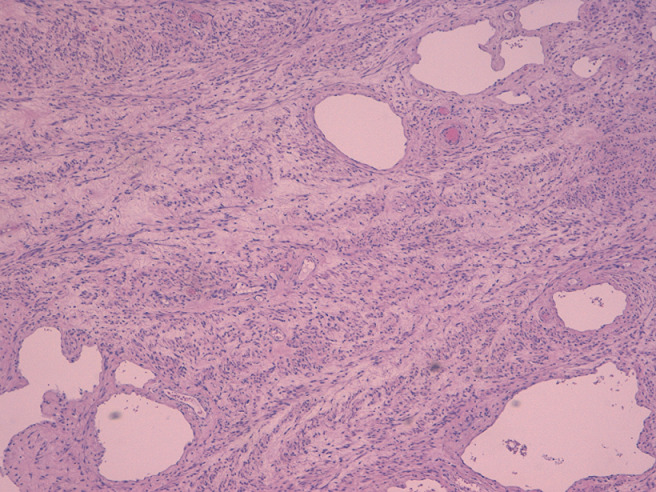
uterine leiomyoma with multiple ectatic vessels and edematous areas, hematoxylin and eosin, 400x

**Follow-up and patient perspective**: her post-surgery hemoglobin was 8.7 gr/dL and the hematocrit was 28%. The patient and her baby were dismissed from the hospital after 4 days, in good clinical conditions, relieved and satisfied with her experience. On histologic examination of the uterus, multiple necrotic and hemorrhagic foci were identified at implantation site of the tumor; the mass was described as a “giant” leiomyoma of 6.7 kg and 28 x 11 x 13 centimeters (Figure 3), with extensive areas of edema and inflammation; the tubes were found to be both invaded by inflammatory cells (Figure 4).

**Informed consent**: the patient gave her consent to analysis and description of her case for scientific purposes.

## Discussion

We report a case of an intra-cesarean myomectomy and hysterectomy performed for the exceptional dimensions of an obstructing, infralegamentary leiomyoma, whose impact on uterine morphology and abdominal topography were previously unseen. Given the conditions of the patient at C-section, the substantial absence of fetal and obstetric complications is impressive, as well as the normal fetal growth. In these instances, referral to a tertiary center is the first step for a positive outcome. Adequate counselling and a multidisciplinary evaluation are necessary, with participation of anesthesiologists and obstetricians, both experts in ultrasonography and obstetric/gynecological surgery [4]. In presence of such voluminous lesions, uterine leiomyosarcoma as well as ovarian cancer should be taken into account in the differential diagnosis and if clinical and imaging suspect is high, delivery and definitive diagnosis should be carried out promptly. Evidence on feasibility and safety of myomectomy during pregnancy is so far adectodal [5]; nevertheless, it may be an alternative if diagnosis is made at an earlier gestational age and if fetal growth appears to be restricted by uterine compression and hypoperfusion [6].

During the pre-operative planning, anticipation and preparedness for predictable hemorrhagic and surgical complications are key elements to successful C-sections. In 2012, Huang *et al*. [7] published a case report of an intracesarean myomectomy of a 30 x 25 cm fibromyoma under regional epidural anesthesia. In our opinion, general anesthesia is more appropriate when dealing with exceptionally voluminous masses during C-sections. First of all, given the abdominal expansion of the mass, classic or even “high” epidural anesthesia may not cover the diaphragmatic and possible thoracic manipulations. Moreover, intensive monitoring granted by general anesthesia seems more reasonable considering the high possibility of long operative time, intra-operative hemorrhage and the sudden hemodynamic alterations when a highly vascularized, 6.7 kg mass is suddenly removed from the pelvis. Gravid uterine exteriorization is a complex maneuver and should be carried out only by expert and well-trained obstetricians as it may have complex hemodynamic consequences [8]. However, when the pelvic anatomy is severely compromised by either placental invasion as in cases of placenta accrete spectrum or by the presence of abdominal masses, it allows to avoid placental incision and a better visualization of the bladder and safer fetal extractions. Moreover, when hemorrhagic complications are likely to occur, it allows prompt hemostatic maneuvers. Management of in-labour patients with abdominal masses such as the one described in this report may be extremely dangerous, especially in less equipped centers [9].

## Conclusion

“Giant” leiomyomas are obstetric entities which pose difficult yet fascinating challenges when diagnosed during the final stages of pregnancy; in our opinion, the three most important elements for safe and successful management are referral to a tertiary center with high expertise in obstetric surgery, rigorous multidisciplinary plan and clear and adequate counselling with the patient.
